# T-cell prolymphocytic leukemia, a case report and review of the literature

**DOI:** 10.32604/or.2025.058175

**Published:** 2025-02-28

**Authors:** LUIS MANUEL GONZáLEZ-RODRíGUEZ, LUIS MIGUEL JUáREZ-SALCEDO, JAVIER LOSCERTALES, EVA ARRANZ, JIMENA CANNATA-ORTIZ, JAVIER ORTIZ, MARIA JOSé LóPEZ DE LA OSA, ADRIáN ALEGRE, SAMIR DALIA

**Affiliations:** 1 Hematology Department, La Princesa University Hospital, Madrid, 28006, Spain; 2 Hematology/Oncology, Mercy Clinic Oncology and Hematology–Joplin, Misouri, MO 64804, USA

**Keywords:** T-prolymphocytic leukemia (T-PLL), MTCP1, JAK/STAT, STAT5B, Alemtuzumab

## Abstract

T-prolymphocytic leukemia is a rare and aggressive hematological malignancy characterized by the clonal proliferation of mature lymphoid T-cells. The pathogenesis of T-PLL is closely linked to specific chromosomal abnormalities, primarily involving the proto-oncogene T-cell leukemia/lymphoma 1 gene family. Recent advancements in molecular profiling have identified additional genomic aberrations, including those affecting the Janus kinase/signal transducers and activators of transcription (JAK/STAT) signaling pathway. This case report presents a patient with T-prolymphocytic leukemia whose cytogenetic and molecular analysis revealed a t(X;14)(q28;q11.2) translocation and a *STAT5B* mutation. Here, we aim to review the genetic and molecular underpinnings of T-prolymphocytic leukemia, as well as current treatment options, with a focus on the anti-CD52 monoclonal antibody alemtuzumab and JAK inhibitors. While alemtuzumab followed by allogeneic hematopoietic stem cell transplantation remains the standard of care for eligible patients, its efficacy is limited and many patients are ineligible. Emerging therapeutic approaches, such as JAK/STAT inhibitors, offer promising potential for improving patient outcomes.

## Introduction

Prolymphocytic leukemia is a hematological malignancy characterized by the proliferation of mature lymphoid cells and can derive from either B-lymphocytes (B-PLL) or T-lymphocytes [1]. This rare disease accounts for ~2% of mature lymphoid cell neoplasms, with T-lymphoid leukemia being more common than B-lymphoid leukemia [2]. The disease primarily affects older adults, with a median age of diagnosis >60 years in different series. While research on its incidence and other demographics is limited, data extracted from the National Cancer Database and the Surveillance, Epidemiology, and End Results database have revealed an increase in its incidence in recent years, likely due to improved recognition and diagnostic criteria, with greater prevalence in males and individuals with a history of other neoplasms [3]. T-prolymphocytic leukemia (T-PLL) is an aggressive neoplasm characterized by lymphocytosis, anemia, thrombocytopenia, and hepatosplenomegaly [4]. The diagnosis, staging, and assessment of response to treatment of this pathology are established according to the working consensus of the T-PLL International Study Group (T-PLL-ISG), with the aim of harmonizing research efforts and supporting clinical decision-making [4].

The pathogenesis of T-PLL is primarily driven by overexpression of the oncogene T-cell leukemia/lymphoma 1 (*TCL1*). Inactivation of the ataxia-telangiectasia mutated (*ATM*) gene and chromosomal abnormalities of chromosome 8 are also common [5]. Moreover, recent studies have revealed a complex mutational landscape, identifying recurrent mutations in genes not previously associated with T-PLL. These mutations often affect epigenetic regulators, DNA repair proteins, and the JAK/STAT pathway [6].

Standard first-line treatment for T-PLL typically involves the humanized anti-CD52 monoclonal antibody alemtuzumab. However, randomized studies specifically evaluating the use of alemtuzumab for T-PLL are lacking, and the therapeutic benefit has been extrapolated from small prospective and retrospective series, which suggest response rates of 92%. Unfortunately, responses to alemtuzumab are often transient and disease progression is inevitable. Without consolidation therapy such as allogenic hematopoietic stem cell transplantation (allo-HSCT), disease-free survival (DFS) typically does not exceed one year [4]. Allo-HSCT is considered the only curative treatment option and would be considered for patients who achieve complete remission (CR). However, only 30%–50% of patients with T-PLL are eligible for allo-HSCT due to reduced performance status, other comorbidities, and age [7]. While current treatment options for relapsed T-PLL are limited and lack strong evidence supporting their use, new therapeutic approaches targeting specific molecular alterations are under investigation. In the present clinical case report, we identified the presence of a *STAT5B* mutation through next-generation sequencing (NGS). Given this finding, we focused our review of novel therapeutic options on strategies targeting the JAK/STAT pathway and *STAT5B*, a potential avenue for improving outcomes in patients with this challenging disease.

## Matherial and Methods

A systematic literature search based on a clinical case was performed using PubMed and EMBASE databases to identify articles and studies that assessed the genetic and molecular landscape of T-PLL, current treatment options in patients with T-prolymphocytic leukemia, and emerging therapeutic approaches, such as JAK/STAT inhibitors. The search was restricted to articles and studies published in English and Spanish.

## Clinical Case

Our case was a 64-year-old woman with no relevant medical history or prior infections. She was independent in her daily activities. She reported being completely asymptomatic from a general point of view, with no associated secondary symptoms. At the time of admission to the hematology department, she had recently been diagnosed with right lobar pneumonia. Despite experiencing mild respiratory symptoms and fever, she remained in generally good health.

Laboratory findings demonstrated progressive leukocytosis with lymphocytosis over the past 6 months; specifically, a leukocyte count of 71.47 × 10^9^/L and lymphocytosis of 62.01 × 10^9^/L. Physical examination was unremarkable, with no masses or palpable lymph nodes and no skin lesions. The remaining laboratory parameters were normal (hemoglobin 11.8 g/dL, platelets 149,000/mm^3^, neutrophils 6.76 × 10^9^/L), with normal coagulation and biochemical parameters (normal renal and hepatic profile, ions in range, corrected calcium 9.2 mg/dL, lactate dehydrogenase 303 U/L). Serology for hepatotropic viruses, HIV and HLTV-1 were negative, but positive IgG antibodies to cytomegalovirus (CMV), herpes simplex, and varicella zoster were detected.

**Figure 1:**
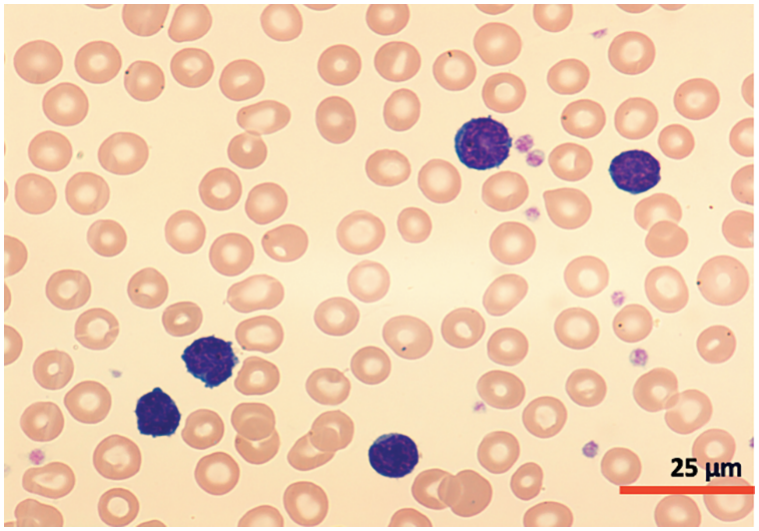
Cytomorphology of leukemic cells from peripheral blood smear. May-Grümwald-Giemsa staining. 40×.

**Figure 2:**
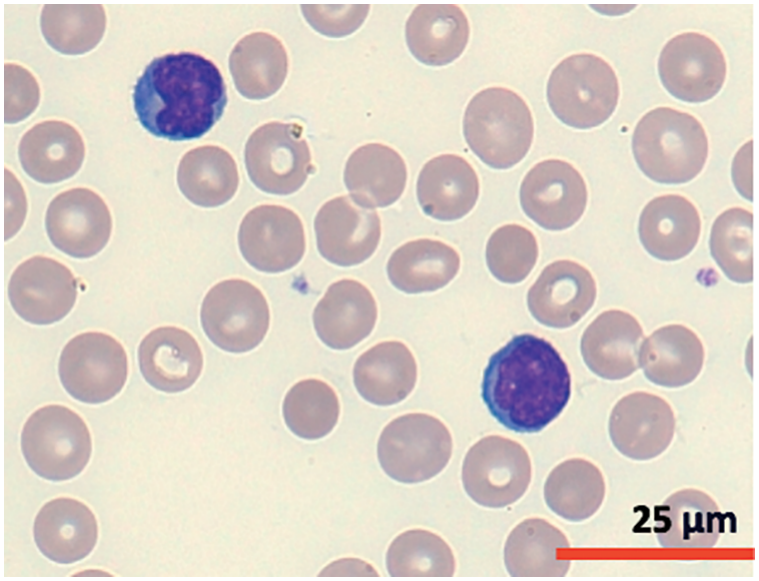
Cytomorphology of leukemic cells from peripheral blood smear. May-Grümwald-Giemsa staining. 100×.

**Figure 3:**
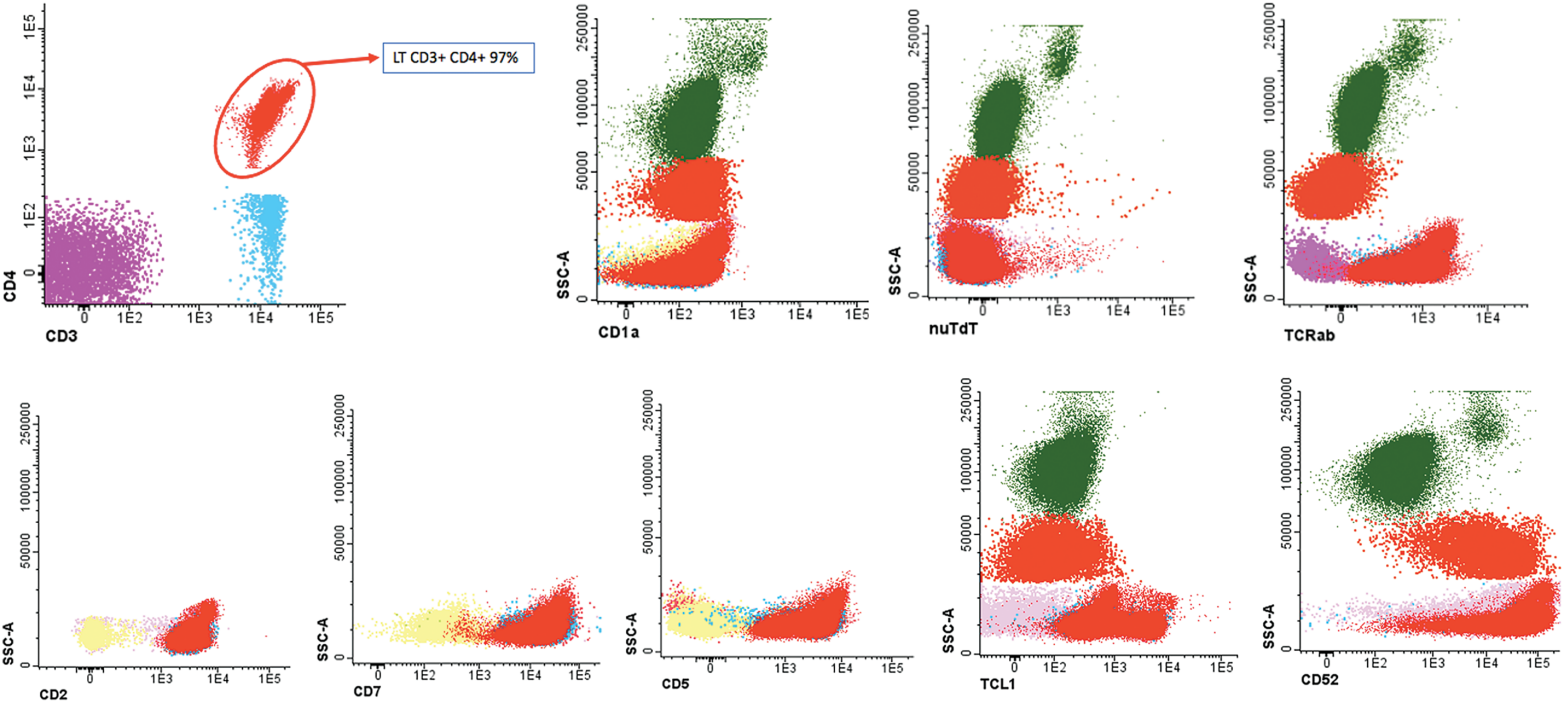
Flow cytometry peripheral blood sample demonstrated an aberrant T-cell population. CD4+ T lymphocytes: 97% of the lymphocyte population (78% of total cellularity) with pathological phenotype: TCRab+, CD34−, CD117, nuTdT−, CD3+, CD4+, CD8−, CD5+, CD45+, CD45RA−, CD7+, CD2+, CD28+, CD26+ and CD27+, CD57−, cyCD30−, CD279+, heterogeneous TCL1 (20%++), CD52++, CD56−, CD1a−, CD10− and CD99−.

A cytomorphological and immunophenotypic study was performed on peripheral blood. Peripheral blood smears (Figs. 1 and 2) confirmed marked lymphocytosis with the presence of small atypical lymphocytes, with condensed chromatin, irregularly shaped nuclei, absence of nucleoli, scant basophilic cytoplasm, and occasional “blebs”. Flow cytometry analysis (Fig. 3) revealed that 78% of lymphocytes were mature T-lymphocytes with a CD4+ immunophenotype, positive for pan-T markers, CD52++, CD26+, TCL1, and exhibiting a T-cell receptor (TCR) rearrangement. These findings are consistent with a diagnosis of T-PLL.

**Figure 4:**
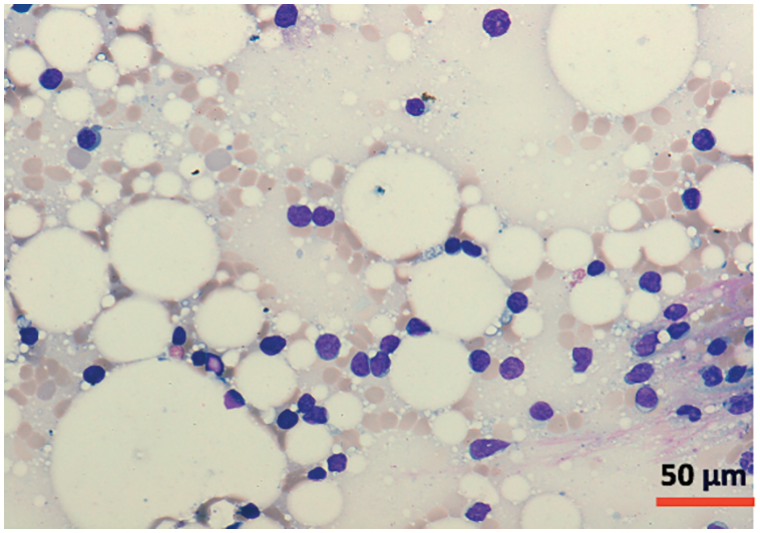
Cytomorphology of leukemic cells from marrow aspirate. May-Grümwald-Giemsa staining. 20×.

**Figure 5:**
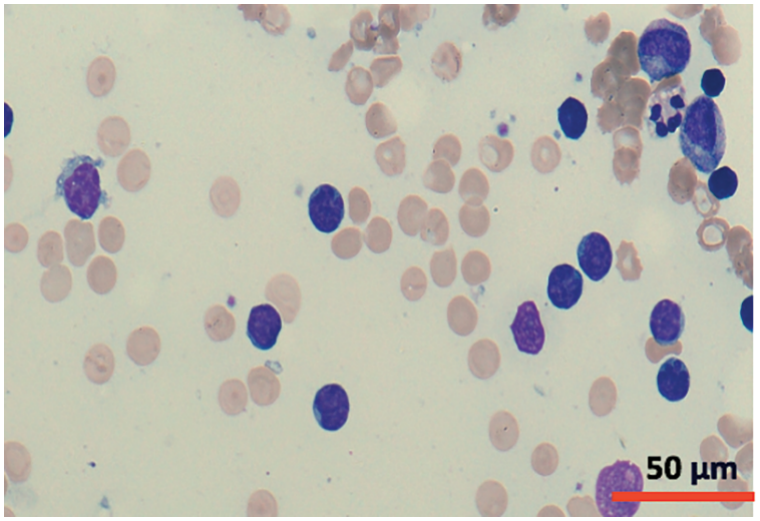
Cytomorphology of leukemic cells from marrow aspirate. May-Grümwald-Giemsa staining. 40×.

**Figure 6:**
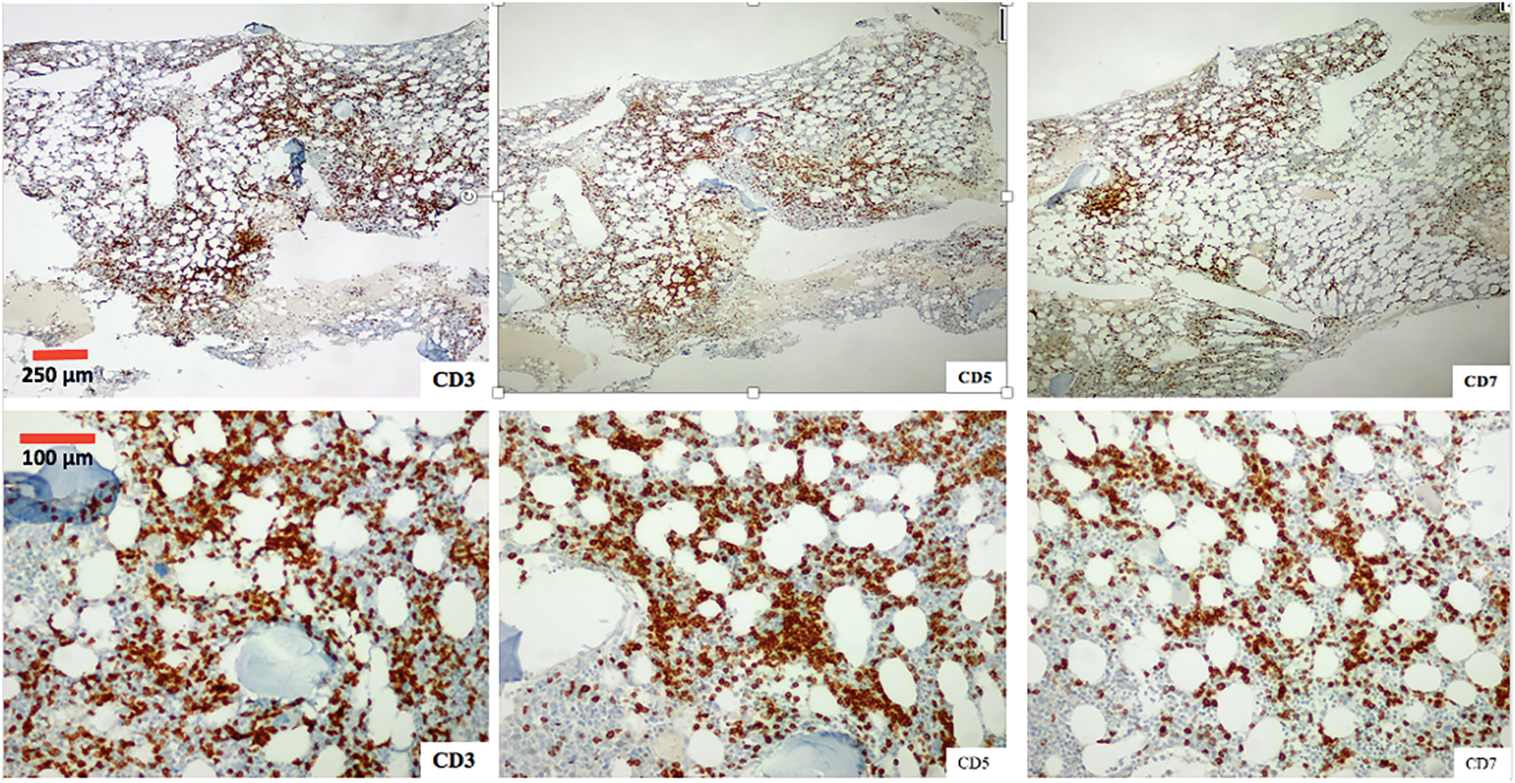
Photomicrograph of the one marrow biopsy, Immunohistochemical staining ×4 (first row) and ×10 (second row) for T-cell markers CD3, CD5 and CD7.

A bone marrow aspirate and biopsy were performed, revealing significant lymphocyte infiltration (50%–60% infiltration; Figs. 4 and 5) with characteristics similar to those described in peripheral blood. The biopsy demonstrated nodular and interstitial lymphocyte accumulations, confirmed by immunohistochemistry as T-cells (positive for CD3, CD5, and CD7) (Fig. 6).

**Figure 7:**
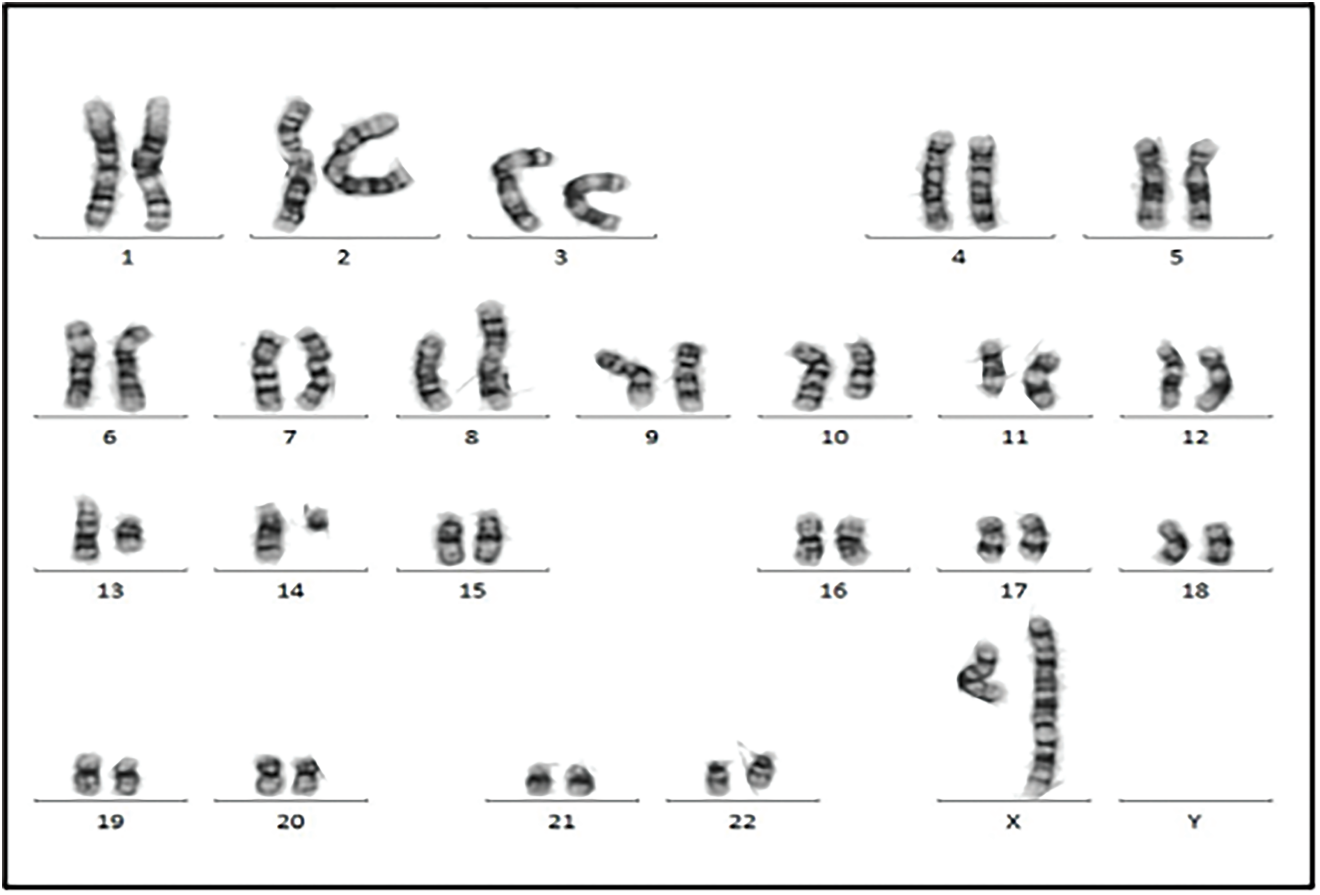
Chromosomal analysis showed the neoplastic cells with a complex karyotype with 46,X, t(X;14)(q28;q11.2),der(X)t(X;14)dup(X)(q26), i(8)(q10), del(10)(q23q34), del(11)(p14p15), del(11)(q14), −13, +mar [10]/46, XX [10].

**Figure 8:**
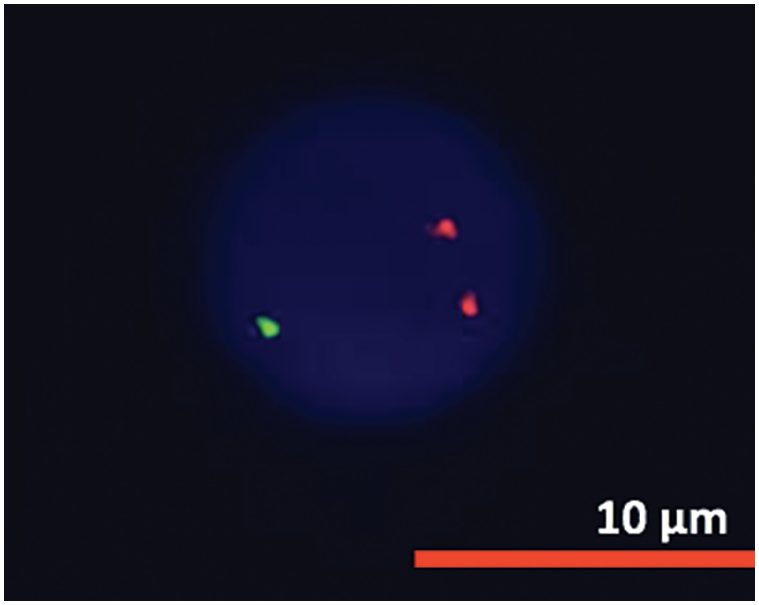
FISH analysis detected ATM deletion.

**Figure 9:**
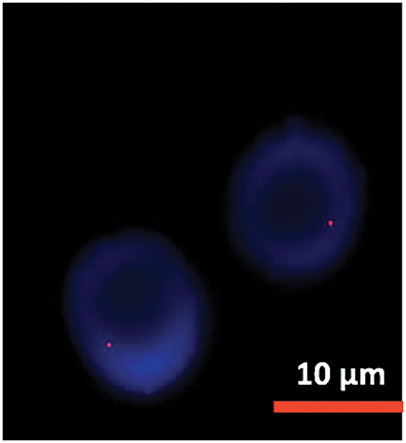
FISH analysis detected 13 deletion.

Cytogenetic analysis (Fig. 7) revealed a complex karyotype with a translocation t(X;14)(q28;q11.2) and a derivative X chromosome, der(X)t(X;14)dup(X)(q26). This chromosomal aberration is associated with disruption of *MTCP1*, a hallmark of T-PLL. Additionally, abnormalities involving chromosome 11 (including the *ATM* gene locus), isochromosome 8, and deletions on chromosome 13 were observed. Fluorescence *in situ* hybridization (FISH) analysis detected T-cell receptor α/δ (TCRA/D) rearrangement and confirmed the deletion of *ATM* and the 13q14.2 region (Figs. 8 and 9). *TCL1a* gene rearrangement was not detected. Due to the unavailability of an MTCP1-specific probe, direct confirmation of the MTCP1 rearrangement by FISH was not possible. NGS of a bone marrow aspirate identified a gain-of-function (GOF) mutation in the *STAT5B* gene (Q706L) with a variant allele frequency (VAF) of 4.9%, considered a pathogenic variant in T-PLL. Furthermore, NGS confirmed the presence of mutations in *ATM*, with a VAF of 51.5%, consistent with a germline origin. Additional mutations were identified in *TYK2* (VAF 39.2%) and *PLCG1* (VAF 1.8%), while no mutations in p53 were detected. Polymerase chain reaction (PCR) analysis of peripheral blood detected clonal TCR gene rearrangements.

A total body computed tomography (CT) scan revealed a normal-sized liver and spleen but identified numerous lymphadenopathies in the cervical, mediastinal, hilar and retroperitoneal regions. While the number of these enlarged lymph nodes was significant, most were not significantly large in size. These findings are consistent with a lymphoproliferative disorder.

Integrating this information with other clinical findings, a diagnosis of T-PLL was established, fulfilling diagnostic criteria. The disease was likely in an indolent phase.

The patient required hospitalization for treatment of the previously described respiratory infection. Despite intravenous antibiotics, her condition deteriorated, leading to sepsis and intensive care unit admission. She developed respiratory failure requiring intubation and mechanical ventilation. Vasoactive support with low-dose noradrenaline was initiated. No specific pathogen was identified.

Given the significant lymphocytosis, nearing 100,000 × 10^9^/L, and the aggressive nature of T-PLL immediate disease burden reduction was necessary. However, due to an ongoing infection, initiating treatment with alemtuzumab was delayed to avoid potential exacerbation. Instead, a pre-phase treatment with corticosteroids and cytoreduction with cyclophosphamide (200 mg/m^2^ for 5 days) was initiated. Despite prophylactic measures with hydration and allopurinol, tumor lysis syndrome developed, necessitating rasburicase administration.

Considering the potential for infectious complications related to alemtuzumab and the temporary control of the disease, we elected to delay the initiation of outpatient treatment until the infection had fully resolved.

No alemtuzumab-related infusion reactions occurred. The patient tolerated the maximum treatment dose well during the first week of administration. Pre-medication with dexchlorpheniramine, paracetamol, and urban was given for all treatment doses. Periodic CMV monitoring by quantitative PCR detected CMV reactivation in blood two days after initiating alemtuzumab treatment, although the patient remained asymptomatic. A peak CMV viral load of 1870 IU/mL was reached five days after treatment initiation. Preventive valganciclovir treatment (900 mg every 12 h for 14 days) was initiated. Alemtuzumab treatment was not interrupted. CMV viral load dropped below 500 IU/mL four days after starting valganciclovir and was undetectable after completing the 14-day course.

The patient tolerated alemtuzumab treatment well, with no significant cytopenias or need for transfusions or G-CSF. As a single infectious complication, the patient developed a new episode of bilateral pneumonia, without microbiological isolation, in the seventh week of alemtuzumab treatment, which resolved after alemtuzumab was temporarily discontinued.

After completing eight weeks of therapy, a CR was confirmed by peripheral blood counts and bone marrow examination. Flow cytometry detected negative minimal residual disease (MRD) in bone marrow. Additional diagnostic tests such as TCR rearrangement and FISH were not performed. A full-body CT scan showed a significant reduction in the lymphadenopathy existing at diagnosis. Given the achieved CR, the patient was considered a suitable candidate for allo-HSCT to consolidate the response.

## Clinical Presentation

T-PLL is typically an aggressive hematological malignancy. Most patients present with a rapidly progressive clinical course characterized by splenomegaly (around 80% of cases, being the most common finding), often massive, secondary symptoms (65%), and lymphadenopathy (50%–60%), usually small in size. Peripheral blood lymphocytosis is usually marked, typically greater than 100,000 × 10^9^/L [2]. T-PLL is also distinguished by the presence of extramedullary involvement. Cutaneous involvement is common (25%) in the form of rash, erythroderma, or nodular lesions, as well as peripheral edema and pleuroperitoneal effusions (15%). Involvement of other extranodal sites such as the lung, kidney, or central nervous system is rare. Hematological cytopenias, particularly anemia, and thrombopenia, are common [2,4].

Approximately one-third of T-PLL cases are initially diagnosed as isolated stable lymphocytosis, a condition characterized by a subtle increase in lymphocyte count without overt symptoms [4]. This form of presentation may lead to misdiagnosis due to similarities with other lymphoproliferative disorders typically associated with stable lymphocyte counts and lack of symptoms, such as chronic lymphocytic leukemia (CLL). All patients diagnosed in this indolent phase will eventually progress to the characteristic aggressive form of the disease.

Notably, there are no discernable clinical, laboratory, demographic, morphological, or immunophenotypic features between the indolent and aggressive forms of the disease at diagnosis. Similarly, there are no clinical or laboratory distinctions between the presentation of secondary progression and initially active forms. T-PLL appears to be a biphasic disease, with the initial indolent phase likely being underdiagnosed.

Awareness of the two disease presentations is essential for optimal therapeutic management. Active disease is indicated by the presence of symptomatic hepatosplenomegaly or lymphadenopathy, secondary symptoms, organ involvement (skin, lung, pleural/peritoneal effusions), or cytopenias, and necessitates immediate treatment. For asymptomatic disease, regular monitoring is crucial to identify the onset of disease-related signs and symptoms, assess changes in blood cell counts, particularly lymphocytosis and cytopenias, and recognize rapid increases in lymphocyte count, similar to B-CLL. A doubling of the lymphocyte count within 6 months, or a 50% increase within 2 months, should prompt treatment consideration [4].

## Diagnostic Criteria

A diagnosis of T-PLL can typically be established through the analysis of peripheral blood samples, integrating morphological and immunophenotypic findings. However, molecular and genetic information, including FISH and chromosome banding analysis, is now essential for definitive diagnosis, particularly in light of recent diagnostic criteria [4]. While NGS is not mandatory, it can provide valuable supplementary information when available. Table 1 outlines the diagnostic criteria for T-PLL. A diagnosis of T-PLL requires the demonstration of the first three major diagnostic criteria. The initial two major criteria are considered defining characteristics of the disease. While the third major criterion, involving alterations in *TCL1a* or *MTCP1*, is prevalent in most T-PLL cases, it is not universally present [4].

**Table 1:** Diagnostic criteria for T-PLL

Major criteria	Minor criteria
Identification of population (>5 × 10^9^) of T-prolymphocytes in peripheral blood or bone marrow. Demonstration of T-lymphocyte clonality by flow cytometry or PCR. Chromosomal rearrangements involving chromosome 14q32 or Xq28; transcriptionally activating, respectively, the TCL1a gene and MTCP1.	Abnormalities affecting chromosome 11 (11q22.3; ATM). Abnormalities on chromosome 8: idic(8)(p11), t(8;8), trisomy 8q. Abnormalities on chromosome 5, 12, 13, 22 or complex karyotype. Involvement of typical T-PLL sites (e.g., splenomegaly, pleuroperitoneal effusion).

The incorporation of molecular and genetic features into diagnostic criteria has advanced the classification of T-cell neoplasms with leukemic features, which often exhibit overlapping clinical and phenotypic characteristics. Patients presenting with mature T-cell leukemias who do not meet the established diagnostic criteria for any of these entities, by definition should be classified as having peripheral T-cell lymphoma, not otherwise specified (PTCL-NOS) [1,8]. Differentiating between T-PLL-TCL1-negative cases and PCLT-NOS with leukemic involvement can be complex. In such cases, the presence of extranodal involvement characteristic of T-PLL and other T-PLL-related genetic alterations can guide the diagnosis. For a definitive diagnosis of T-PLL/TCL1-negative cases, the presence of one of these minor diagnostic criteria is necessary [4].

While evaluation of bone marrow infiltration is standard practice in the initial assessment of lymphoproliferative processes, it is not necessary to definitively diagnose T-lymphoproliferative leukemia. However, it remains important for evaluating treatment response. To differentiate between T-lymphoproliferative leukemia and adult T-cell leukemia/lymphoma (A-TLL), serological testing or negative PCR for human T-lymphotropic virus 1 (HTLV-1) is required.

## Cytomorphology

Three different morphological variants of neoplastic cells have been described in T-PLL, the characteristics of each of these forms are presented in Table 2 [2].

**Table 2:** Cytological features of the cellular variants of T-prolymphocytic leukaemia

Variant	Cell size	Nucleolus	Nucleus	Nucleoplasm to cytoplasm ratio
**Classical (75%)**	Medium	Yes	Irregular	High
**Small cell (20%)**	Small	No	Irregular	High
**Cerebriform (5%)**	Medium	Yes	Cerebriform	High

Typical prolymphocytes in T-PLL are medium-sized cells characterized by a condensed nuclear chromatin pattern, a prominent nucleolus, a high nuclear-to-cytoplasmic ratio, and a basophilic, non-granular cytoplasm with occasional small cytoplasmic protrusions or blebs [2].

## Flow Cytometric Immunophenotype

Prolymphocytes characteristic of T-PLL have a distinctive immunophenotype typical of a post-thymic mature T-cell neoplasm, lacking TdT and CD1a expression while exhibiting positivity for CD3. The majority of T-PLL cells display a CD4+ CD8− phenotype, with less frequent CD4+ CD8+ co-expression and rare other CD4/CD8 combinations. Pan-T markers, including CD2, CD5, and CD7, are typically expressed. CD26 is positive, and CD25 is present in ~25% of cases. A defining feature of T-PLL is the universal expression of CD52, which serves as the therapeutic target of the monoclonal antibody alemtuzumab. There is an absence of natural killer (NK) cell markers. The definitive diagnosis of T-PLL is confirmed by the detection of TCL1 [9,10]. While the anti-human TCL1 monoclonal antibody (BD Pharmigen Alexa Fluor) employed in our panel detects TCL1, it cannot differentiate between TCL1a and MTCP1, likely due to their structural homology.

## Genetics and Molecular Profile

T-PLL is usually associated with complex karyotypes (70%–80% of cases). The most common and specific cytogenetic abnormality is the rearrangement of the *TCL1* gene (more than 90% of cases), which is considered the primary event in T-PLL pathogenesis. The *TCL1* gene family of proto-oncogenes consists of 5 isoforms, with *TCL1a* and *MTCP1* (mature T-cell proliferation) being the more relevant for T-PLL. These isoforms are located on chromosome 14q32.1 and Xq28.1, respectively. In most cases, inv(14)(q11.2q32) or t(14;14) (q11.2;q32), juxtapose *TCL1a* with the *TCR A/D* locus, leading to overexpression of *TCL1a* through positive regulation by TCR enhancing elements. Rarely, as in the case we report, t(X;14)(q28;q11.2) juxtaposes the *TRA/D* locus at 14q11.2 with *MTCP1* at Xq28, resulting in overexpression of the MTCP1 oncoprotein [5]. To date, only 15 isolated cases of T-PLL with t(X;14)(q28;q11.2) have been described [11]. The clinicopathological features and molecular profile of patients with t(X;14)(q28;q11.2) T-PLL are not well characterized. It remains unclear whether they share similar characteristics with classical T-PLL with 14q32/TCL1a gene rearrangement. TCL1 and MTCP1 proteins present similarity in a large part of the amino acid sequence [5].

TCL1 acts as a cofactor for Akt kinase. TCL1 is involved in lymphopoiesis and is normally expressed only in immature lymphoid cells. Overexpression in mature lymphoid cells is abnormal [5].

Inactivation of the *ATM* gene is found in more than 80% of cases, and germline mutations in *ATM* increase the risk of developing T-PLL. ATM is a tumor suppressor, and changes in its expression disrupt DNA repair [5]. While deletions or mutations in p53 are not highly prevalent, a functional alteration in p53 often occurs due to the loss of ATM [7]. These findings may contribute to the chemorefractory nature of T-PLL.

Other recurrent genetic changes include abnormalities on chromosomes 8, 5, 12, 13, 22 [5]. No individual cytogenetic abnormality has prognostic implications, but complex karyotypes are associated with worse outcome [12].

While the previously described alterations in *TCL1* are not sufficient to induce leukemogenesis, a second mutational impact model has been proposed for clonal evolution in T-PLL [13]. Recent studies have expanded our understanding of the mutational landscape beyond previously identified lesions. A meta-analysis of 10 published studies (n = 275) revealed constitutive hyperactivation of *STAT5B* in all analyzed samples. In 90% of these cases, a known genomic alteration could explain this hyperactivation, suggesting the identification of a new molecular signature. Furthermore, 62.1% of the cases harbored somatic mutations related to GOF, most frequently in *JAK3* (36.4%), *STAT5B* (18.8%), and *JAK1* (6.3%), often as a subclonal event. More than two-thirds of the other cases without JAK/STAT mutations showed alterations in negative regulators, which could also explain the overactivation [14]. These findings align with previously published series [6,15,16]. Other common genetic lesions include *EZH2* (epigenetic regulators), and *CHEK2* (DNA repair/checkpoint) [6].

Based on these findings, a model of constitutive activation of the JAK/STAT pathway in the pathogenesis of T-PLL has been proposed. The JAK/STAT pathway is involved in lymphopoiesis, participating in the proliferation, differentiation, and migration of T-cells.

In the previously mentioned series of 15 T-PLL/MCTP1 patients, all patients with available NGS molecular studies had mutations in the JAK/STAT pathway, with JAK3 mutations being the most common [11].

## Differential Diagnosis

T-PLL must be distinguished from other mature T-cell lymphoproliferative neoplasms that can manifest in the peripheral blood, including large granular lymphocyte (LGL) leukemia, Sézary syndrome (SS), A-TLL, and aggressive NK-cell leukemia. The integration of clinical, cytomorphological, immunophenotypic, and genetic information is crucial for a correct diagnosis [10,17]. Table 3 provides a summary of the main differential characteristics.

**Table 3:** Differential diagnosis of T-PLL with other mature T-cell lymphoproliferative neoplasms with leukaemic expression

T-cell large granular lymphocyte [18]	Autoimmune diseases, particularly rheumatoid arthritis.
	Asymptomatic. Recurrent infections, frequent neutropenia (50%). Infrequent lymphadenopathy.
	Abundant cytoplasm with azurophilic granules. Eccentric nucleus with dense chromatin.
	CD3+, CD2+, CD8+/CD4− CD27−, CD28−, CD5−, CD7−, CD57+, CD16+, CD45RA+, CD45RO−, TCRαβ+, CD56−, KIR (CD158)+.
**Sezary syndrome** [19]	Classic triad: erythroderma, generalised lymphadenopathy, Sézary cells in peripheral blood.
	Sézary cells. Medium to large size, large, round nucleus with overlapping folds resembling brain convolutions.
	CD3+, CD4+/CD8−, CD5+, CD7−, CD26−, CD25−, CCR4+, CD158k+.
**Adult T-cell lymphoma leukaemia** [20]	HLTV-1+, Japan, Caribbean islands, South America, parts of Africa.
	Acute form (most common). Aggressive behavior. Hypercalcaemia. Skin lesions, lymphadenopathy, hepatosplenomegaly.
	Flower cells. Medium to large size, scant cytoplasm, irregular polylobulated nucleus with flower-like outline.
	CD3+, CD2+, CD4+/CD8−, CD5+, CD7−, CD26−, CD25++, FOXP3+, CCR4+.
**Aggressive NK-cell leukaemia** [21]	Very aggressive behavior. Coagulopathy, multi-organ failure. Haemophagocytic syndrome.
	Similar to LGL, size usually superior, occasionally blast-like appearance.
	CD3-, CD2+, CD4-/CD8−/+, CD56+, CD94+, CD57−, CD16+.

A diagnosis of A-TLL requires a positive HTLV-1 serology. While these T-cell neoplasms share some clinical features, certain characteristics can help distinguish them. The presence of hepatosplenomegaly at diagnosis is more common in A-TLL and LGL leukemia, while palpable lymphadenopathy is typical in SS. Skin lesions are common across all entities, although the presence of generalized erythroderma is characteristic of SS. T-PLL often presents with a rapid increase in lymphocyte count. Eosinophilia is common in SS, while neutropenia is characteristic of LGL leukemia. Autoimmune phenomena are frequent in LGL leukemia [8]. Among T-cell neoplasms with peripheral blood expression, T-PLL is the only entity with a uniformly defined molecular abnormality used for diagnostic purposes.

## First-Line Treatment in T-PLL

Patients with indolent forms of disease, irrespective of treatment, inevitably progress to aggressive disease over time. No statistically significant difference in the rate of this progression has been observed between treated and untreated patients. Therefore, there is currently no evidence to support the immediate initiation of treatment for indolent disease. The decision to start treatment is typically based on the presence of active disease [4].

Conventional chemotherapy has proven ineffective in treating T-PLL. CHOP (cyclophosphamide, doxorubicin, vincristine, prednisone) based and related regimens have been disappointing [4]. Alternative regimens have also been tested in T-PLL. For instance, intravenous pentostatin monotherapy achieved medinan DFS of only 6 months [4]. While intravenous bendamustine monotherapy showed some promise, with a 55.3% PR rate and improved DFS and overall survival (OS) for responders, the median OS for all patients remained below 9 months [22]. Given these underwhelming outcomes, chemotherapy is not considered a first-line treatment for T-PLL [2].

Intravenous alemtuzumab is the most effective treatment for patients with T-PLL. Dearden et al. described CR rates of up to 81% with alemtuzumab monotherapy in previously untreated patients, with a 12-month DFS of 67 [23]. Unlike chronic lymphoid processes, subcutaneous administration of alemtuzumab was considerably less effective than intravenous administration, even compared with relapsed/refractory patients treated with intravenous alemtuzumab beyond the first-line setting [23]. In contrast to chronic lymphoid processes where polychemotherapy regimens such as fludarabine, cyclophosphamide, and mitoxantrone can enhance treatment outcomes, using this regimen before alemtuzumab did not improve the overall response rate (ORR) or DFS [24], and could even increase toxicity, potentially hindering the completion of full intravenous alemtuzumab treatment [25].

While the combination of alemtuzumab with pentostatin yielded superior results to those obtained with pentostatin alone, as expected from the data presented above, it did not demonstrate statistically significant improvements in depth or duration of response (CR: 62%, DFS: 7.8 months in pre-treated patients) [26] compared with intravenous alemtuzumab monotherapy, as reported by Deaden et al. [23]. However, the combination did outperform the regimen used by Keating et al. [27] in pre-treated patients with manageable toxicity. Intravenous alemtuzumab monotherapy regimens showed significantly higher DFS and OS than combination regimens with pentostatin, both in untreated and pre-treated patients [28]. No prospective studies have directly compared alemtuzumab in monotherapy with its combination with pentostatin. Additionally, there is no evidence supporting the benefit of alemtuzumab maintenance therapy, hence it is not recommended [4]. Table 4 summarizes the results of the above-mentioned studies.

**Table 4:** Summary of first-line treatment studies on T-PLL

Treatment regimen	Authors	Clinical trial	Disease status	n	ORR (%)	CR (%)	PR (%)	DFS (months)	OS (months)
Alemtuzumab IV	Dearden et al. [4]	Multicentre, prospective	Pre-treated	39	76	60	16	7	10
Alemtuzumab IV	Keating et al. [27]	Multicentre, retrospective	Untreated	4	75	75	0	4.5	7.5
			Pre-treated	72	50	37.5	12.5		
Pentostatina + alemtuzumab IV	Ravandi et al. [26]	Unicentric, retrospective	Pre-treated	13	69	62	8	7.8	10.2
Alemtuzumab IV	Dearden et al. [23]	Unicentric, prospetive	Untreated	32	91	81	10	12	48
Alentuzumab SC			Untreated	9	33	33	0	12	48
Alemtuzumab IV			Pre-treated	45	74	60	14	12	48
FMC + consolidation with alemtuzumab IV	Hopfinger et al. [25]	Multicentre, prospective	Untreated	16	92	48	44	11.5	17.1
			Treated	9					
Bendamustine	Herbaux et al. [22]	Multicentre, retrospective	Untreated	6	55.3	20	33.3	5	8.7
			Pre-treated	9					
Alemtuzumab IV	Jain et al. [28]	Unicentric, retrospective	Untreated	42	81	61	20	11	15
Alemtuzumab IV + pentostatin			Untreated	13	82	73	9	4.3	10.4
Alemtuzumab IV			Pre-treated	15	46	46	–	3	15
Alemtuzumab IV + pentostatin			Pre-treated	5	75	50	25	2.6	2.6

Note: ORR, overall response rate; CR, complete remission; PR, partial respone; DFS, disease free survival; OS, overall survival; IV, intravenous; SC, subcutaneous.

*A. Highlights of treatment with alemtuzumab*

Alemtuzumab, a humanized IgG1 kappa monoclonal antibody targeting the CD52 antigen, has been used to treat B-CLL. Indeed, most of the safety and adverse event data for alemtuzumab come from studies on patients with CLL. CD52 is a cell surface glycoprotein highly expressed on the surface of various immune cells, including normal and neoplastic lymphocytes, monocytes, macrophages, and eosinophils. It is absent on hematopoietic precursors, red blood cells, and platelets [2]. Alemtuzumab binds to CD52, triggering cell lysis through complement-dependent cytotoxicity and antibody-dependent cytotoxicity, leading to lymphodepletion.

The dosing and duration of alemtuzumab treatment are outlined in Table 5 [2]. Peak drug doses are usually achieved within the first week of treatment. Infusion-related adverse reactions during early treatment might necessitate slower dose escalation. If treatment is interrupted for more than one week, the dose should be gradually increased upon restarting therapy.

**Table 5:** Alemtuzumab administration protocol

Dosage	3 times/week, every other day. Intravenous (IV) administration.
	First week of treatment: increasing doses. Day 1, first dose: 3 mg. Day 3, second dose: 10 mg. Day 5, third dose: 30 mg.
	In case of tolerance, maintain maximum dose of 30 mg until end of treatment.
	Dilution in 100 mL of sodium chloride 0.9%. Administration by intravenous infusion over 2 h is proposed.
**Duration of treatment**	Maximun 12 weeks of treatment.
	Until complete response criteria are met.
**Premedication**	Paracetamol 1 g (oral or intravenous) + dexchlorferniramine 5 mg (oral or intravenous), 30–60 min before infusion.
	During dose escalation, and in patients with previous severe infusion reactions, intravenous methylhydrocortisolone 40mg may be considered. Do not routinely administer corticosteroids, discontinue after the first week of treatment.
	In case of development of severe infusional reaction: discontinue treatment immediately.

The duration of alemtuzumab treatment is determined by the patient’s response. If disease progression or lack of response occurs during treatment, discontinuation should be considered. Consensus guidelines recommend a bone marrow evaluation to confirm CR [4]. Alemtuzumab treatment should continue until the bone marrow is disease-free. Once CR is achieved in the peripheral blood and in affected nodal and extranodal sites, a bone marrow aspirate and biopsy should be considered.

The criteria for CR are outlined in Table 6, requiring the fulfillment of all specified parameters [4]. While there are no specific criteria for assessing remission of nodal involvement in T-PLL, the RECIL criteria [29] are proposed due to their adequate correlation in response assessment between unidimensional and bidirectional measurement. Alternatively, the Lugano [30] and iwCLL [31] criteria can be considered [4].

**Table 6:** CR criteria after treatment with alemtuzumab in T-PLL

Longitudinal diameter of lymphadenopathies described at diagnosis less than 1 cm, by CT or MRI.
Absence of hepatosplenomegaly, by imaging test.
Absence of secondary symptoms and disease-related symptoms.
Circulating lymphocyte count in peripheral blood less than 4 × 10^9^/L.
Pathological population in bone marrow less than 5% of total nucleated cells, by immunohistochemistry or flow cytometry.
Absence of disease in other affected organs or tissues.
Absence of cytopenias (platelets >100,000 × 109/L, haemoglobin >11.0g/dL, neutrophils >1.5 × 109/L).

Currently, CR assessment does not require the use of MRD assays. Despite the genetic nature of T-PLL, molecular biology and genetics do not play a significant role in routine response assessment. The clinical relevance of MRD assessment in T-PLL remains undefined. However, in parallel with standardized flow cytometry, techniques such as TCR rearrangement analysis and FISH could be applied to assess the absence of diagnostic genetic alterations, providing insights into treatment response depth, particularly in clinical trial settings.

*B. Infectious risk*

Alemtuzumab significantly weakens the immune system, posing a serious risk of opportunistic infections in patients already immunocompromised due to their underlying condition. Prevention and proper management of infectious complications are thus crucial.

Prophylactic measures with co-trimoxazole and acyclovir are recommended [2]. Importantly, the recovery of CD4+ cell counts may be delayed for several months after the last dose of alemtuzumab. Additionally, there might be a prolonged period of immunodeficiency even after normal lymphocyte counts return. Therefore, a conservative approach suggests continuing both prophylaxes for six months post-treatment or until CD4+ cell counts are above 250,000/μL. Antifungal and antibacterial prophylaxis is not recommended [2].

Bacterial infections occur predominantly at the beginning of alemtuzumab treatment. Viral infections tend to start around three weeks into treatment, and fungal infections become more frequent after one month of treatment.

Immunocompromised patients are at increased risk of CMV infection. Indeed, CMV reactivation is considered the most common opportunistic infection associated with alemtuzumab therapy in this patient group. While preemptive therapy significantly reduces the risk of developing CMV disease, it is not entirely eliminated. A retrospective Spanish study on patients with T-PLL reported an incidence of CMV reactivation of 38.9% and CMV disease of 4.9% [32]. Early studies likely underestimated these figures due to limited CMV monitoring capabilities. By contrast, recent studies, that incorporate periodic CMV viral load monitoring and proactive preventive measures, report lower rates of symptomatic infections.

CMV reactivation does not occur after the completion of alemtuzumab therapy. Patients receiving concurrent steroid therapy were found to be at a heightened risk of CMV reactivation [32].

A CMV serological test is crucial before initiating treatment. While specific CMV prophylactic treatment can prevent symptomatic reactivation, it is not routinely recommended. However, it may be considered for high-risk patients at the discretion of the healthcare provider. Regular monitoring of CMV viral load through quantitative PCR is advised. Asymptomatic patients with positive CMV viremia should receive preemptive treatment but can continue alemtuzumab therapy. Discontinuation of alemtuzumab should be considered in patients experiencing CMV symptoms or diagnosed with CMV disease [33].

Patients treated with alemtuzumab are susceptible to a wide range of infections. It is imperative to assess the patient’s serological status and history of previous infectious processes to enable effective surveillance and prevention of reactivation of latent infections.

## Role of Allogeneic Hematopoietic Stem Cell Transplantation in T-PLL

Allo-HSCT is currently the only potentially curative therapy and should be considered in eligible patients after achieving a response to cytoreductive therapy [34]. However, it has been published that in the majority of patients who achieved complete remission, this procedure was not associated with increased progression-free survival or overall survival [28]. A multi-institutional retrospective case series of 27 T-PLL patients, reported on behalf of the French Society for Stem Cell Transplantation (SFGM-TC), showed 3-year DFS and OS after allo-HSCT of 26% and 36%, respectively [35]. Another study evaluating data from CIBMTR (Center for International Blood and Marrow Transplant Research) reported 4-year rates of OS and DFS of 30% and 25.7% with allo-HSCT [36]. A retrospective study from the European Society for Blood and Marrow Transplantation (EBMT) in 41 patients who received allo-HSCT reported a 3-year relapse-free mortality (NRM) of 41%, DFS 19%, and OS 21% [37]. Allo-HSCT has been reported to yeld durable remissions, especially if offered after achieving a CR [38].

Disease relapse is the most frequent cause of death (about 50% of cases) in these patients [28]. In some cases, if allogeneic transplantation is not possible, autologous transplantation may be considered.

## New Therapies in the Treatment of T-PLL

A deeper understanding of T-PLL pathogenesis has opened the door to novel therapeutic strategies targeting specific molecular pathways. Some of these approaches are currently being investigated in clinical trials [39].

Approaches include MDM2 inhibition to restore p53 activity in T-PLL cells and induce apoptosis; PARP inhibition to promote apoptosis by impairing DNA repair mechanisms; and BH3 mimetics to target the Bcl-2 family of proteins and induce apoptosis. Another approach is epigenetic therapy such as hypomethylating agents and HDAC inhibitors to exploit the high frequency of epigenetic alterations. Because positive regulation by TCR enhancer elements is involved in the pathogenesis of T-PLL, TCR kinase inhibition (ITK inhibitors) as well as AKT/PI3H inhibitors would block TCR signaling pathways [10,17]. Furthermore, the development of novel antibodies with reduced immunosuppressive effects is a priority. CCR7, a prognostic biomarker for OS in T-PLL, is a promising target [40]. While chimeric antigen receptor T-cell therapy offers potential, the identification of specific antigens on malignant T-cells remains a significant challenge, limiting its current application in T-PLL.

GOF mutations involving JAK/STAT family members, recurrent in T-PLL, together with the successful use of JAK/STAT inhibitors in myeloproliferative neoplasms and graft-versus-host disease, suggest that JAK inhibition may offer new therapeutic avenues for T-PLL [41].

*Ex vivo* drug screening has revealed the sensitivity of neoplastic T-PLL cells to JAK inhibitors, even in cases where this sensitivity is not directly predicted by the specific mutational status of JAK/STAT [16]. While JAK3 mutations have been linked to lower rates of OS, neither *STAT5B* mutations nor other mutations in GOF genes appear to have prognostic implications [16,42]. The *STAT5B* N642H, a highly activating mutation of the pathway, appears to confer resistance to ruxolitinib [16].

A few isolated studies have explored the use of JAK inhibitors in patients with T-PLL. The results of these studies are summarized in Table 7.

**Table 7:** Summary of clinical investigations with JAK inhibitors on T-PLL

Treatment regimen	Authors	Study type	Disease status		n	ORR (%)	CR (%)	PR (%)	DFS (months)	OS (months)
Alemtuzumab IV + Itacitinib	Tapan M. Kadia et al. [43]	Phase I clinical trial	Pre-treated		7	50	33	17	7.8	8.5
			Untreated		6	100	50	50	11.7	NR
Venetoclax + rulotinib	Herbaux et al. [44]	Multicentre, prospective	Pre-treated	WT	3	33.3	0	33.3	1.8	NA
				JAK mutated	12	83.3	0	83.3	5.6	
Ventoclax + ruxolitnib	Brothers et al. [45]	Case report	Pre-treated		Patient achieved partial response.					
Tofacitinib + ruxolitinib	Gomez-Arteaga et al. [46]	Case report	Pre-treated		Patient had 10 moths of partial response.					

Note: ORR, overall response rate; CR, complete remission; PR, partial respone; DFS, disease free survival; OS, overall survival; IV, intravenous; NR, not reached; NA, not available

A phase I clinical trial (NCT03989466) assessed the combination of alemtuzumab with itacitinib (JAK1 inhibitor). This trial, the largest to date utilizing JAK inhibitors in T-PLL, demonstrated promising results. In pre-treated patients, the ORR was 50%. When used as a front-line treatment, all patients achieved at least a PR. Moreover, the CR rate was 30% in pre-treated patients and 50% in untreated patients. These CRs were associated with deep remissions, with negative MRD by flow cytometry. The median first-line DFS was 11.7 months, and the median OS had not been reached at 16 months (with OS exceeding 70% at six months). The addition of the JAK inhibitor did not increase toxicity [43].

Inhibition of the JAK/STAT pathway with ruxolitinib increases the dependence of neoplastic cell survival on the Bcl-2 protein [47], thereby sensitizing T-PLL cells to venetoclax. This suggests that the combination of these two agents could yield improved outcomes. A retrospective multicenter study involving 15 patients with refractory/relapsed disease evaluated the combination of ruxolitinib and venetoclax. The ORR was 73.3%, with only PRs observed. Analysis of the molecular status revealed better outcomes in patients with JAK mutations, as the median progression-free survival was significantly shorter in the wild-type group (5.6 months vs. 1.8 months) [44].

A preclinical study using a combination of ruxolitinib and tofacitinib demonstrated an additive effect *ex vivo* [46]. Additionally, a clinical case report described a patient with refractory T-PLL treated with this combination who achieved a PR that was sustained for ten months [46]. Finally, the selective STAT-5 inhibitor pimozide has proven effective in cultured T-cells [6].

## Discussion

The present case report highlights a rare cytogenetic anomaly in T-PLL, with isolated cases described in the literature [11]. Cytogenetic findings revealed the rearrangement of Xq28 (where *MTCP1* is located) juxtaposed with the *TCR A/D* locus on chromosome 14. Unlike most cases of T-PLL, *TCL1a* is not rearranged (by FISH). TCL1 overexpression is detected by flow cytometry, and may occur in both MTCP1/T-PLL and TCL1a/T-PLL due to the high structural homology between these proteins of the same proto-oncogene family. The clinical, pathological, immunophenotypic, and genetic profiles of patients with t(X;14)(q28;q11.2) are not well characterized due to the rarity of the disease and the lack of overlap data with TCL1a/T-PLL [11]. The low incidence of cases limits progress, necessitating further data collection.

As in other hematological diseases, cytogenetic and molecular knowledge plays a crucial role and is already integrated into new T-PLL definitions [4]. While FISH and chromosome banding analysis are essential, NGS, though not currently required for diagnosis, provides valuable additional information. As molecularly defined prognostic subgroups and targeted therapies emerge, NGS may soon influence treatment decisions.

Alemtuzumab, an anti-CD52 monoclonal antibody, is the best available first-line therapeutic option. Its long history of use has led to the development of well-established treatment protocols to manage associated complications. However, the responses are temporary, and early relapse is common, posing a significant challenge. Cures are primarily achieved through allo-HSCT, but this is not always feasible for all patients. Given the poor prognosis and the lack of approved drugs, there is an urgent need for novel therapies, especially in relapsed/refractory disease. Recent advancements in understanding the molecular basis of T-PLL [6] and the identification of new target molecules [7,10,16] offer hope for future treatments. Clinical trial development is hindered by the low incidence of the disease, emphasizing the importance of case reporting.

In our case, NGS upon diagnosis identified a *STAT5B* mutation, aligning with the JAK/STAT pathway and the mutational profile of T-PLL observed in recent studies [6,11,14,15,42]. Given this specific molecular target, we focused our attention on potential therapeutic options targeting this pathway. Available studies cannot be directly compared limited by factors such as population design and sample size, but it suggests that adding JAK inhibitors to alemtuzumab may not significantly improve response rates compared with monotherapy, they may offer potential benefits in terms of DFS and OS, which is valuable. However, randomized controlled trials are necessary to definitively establish the efficacy of this combination therapy, so in the case reported we use alemtuzumab as monotherapy in the frontline. Considering the limited treatment options for relapsed T-PLL and the need to improve first-line outcomes, we propose to continue investigating the addition of a JAK inhibitor as a potential therapeutic strategy. For our patient in the event of relapse, guided by the molecular insights gained from NGS testing, we propose its use.

We need institutions to share data to help better figure out if JAK inhibitors would be useful in the upfront treatment of T-PLL patients with JAK/STAT pathway mutations in conjunction with alemtuzumab. If this can occur, we would propose a clinical trial randomizing patients to alemtuzumab vs. the combination with a JAK inhibitor. This may improve outcomes and even limit bone marrow transplant needs in this poorly understood and deadly disease.

## Conclusions

T-PLL is a rare disease with limited treatment options and a low success rate. Alemtuzumab is currently the treatment with the most promising outcomes. However, new therapies are needed that offer better response rates and fewer side effects. A deeper understanding of the pathogenesis of this disease has identified potential new therapeutic targets, which are now being investigated in various clinical trials. While the initial results of these trials are encouraging, further research is necessary to confirm their efficacy.

## Data Availability

The datasets generated and/or analyzed during the current study are available from the corresponding author upon reasonable request.
